# A Mini Review on Capillary Isoelectric Focusing-Mass Spectrometry for Top-Down Proteomics

**DOI:** 10.3389/fchem.2021.651757

**Published:** 2021-04-09

**Authors:** Tian Xu, Liangliang Sun

**Affiliations:** Department of Chemistry, Michigan State University, East Lansing, MI, United States

**Keywords:** capillary isoelectric focusing-mass spectrometry, top-down proteomics, ESI interface, ampholytes, proteoform, monoclonal antibody, hydrophobic protein

## Abstract

Mass spectrometry (MS)-based top-down proteomics (TDP) requires high-resolution separation of proteoforms before electrospray ionization (ESI)-MS and tandem mass spectrometry (MS/MS). Capillary isoelectric focusing (cIEF)-ESI-MS and MS/MS could be an ideal method for TDP because cIEF can enable separation of proteoforms based on their isoelectric points (pIs) with ultra-high resolution. cIEF-ESI-MS has been well-recognized for protein characterization since 1990s. However, the widespread adoption of cIEF-MS for the characterization of proteoforms had been impeded by several technical challenges, including the lack of highly sensitive and robust ESI interface for coupling cIEF to MS, ESI suppression of analytes from ampholytes, and the requirement of manual operations. In this mini review, we summarize the technical improvements of cIEF-ESI-MS for characterizing proteoforms and highlight some recent applications to hydrophobic proteins, urinary albumin variants, charge variants of monoclonal antibodies, and large-scale TDP of complex proteomes.

## Introduction

Top-down proteomics (TDP) aims to globally characterize proteoforms in cells. The concept of “proteoform” was published in 2013 to describe all the forms of protein molecules derived from a same gene on account of genetic variations, alternative splicing, and post-translational modifications (PTMs) (Smith et al., 2013). As proteoforms from a same gene can have divergent functions, characterization of proteomes in a proteoform-specific manner is imperative for understanding critical biological processes and disease mechanism (Ntai et al., 2018; Smith and Kelleher, 2018). TDP is ideal for characterization of proteoforms because it directly measures intact proteoforms using mass spectrometry (MS) and tandem mass spectrometry (MS/MS) for determining proteoforms' masses, sequences, and PTMs (Chen et al., 2017). Large-scale TDP of complex proteomes require sufficient liquid-phase separations of proteoforms prior to MS and MS/MS (Tran et al., 2011; Chen et al., 2017; Gomes and Yates, 2019; Schaffer et al., 2019; Shen et al., 2019).

Capillary isoelectric focusing (cIEF) separates amphoteric compounds (e.g., proteins) according to their isoelectric points (pIs) with the assistance of ampholytes (Righetti et al., 1997). cIEF can achieve ultrahigh-resolution separation of proteins with as low as 0.004 pI differences (Shen et al., 1999; Kahle and Wätzig, 2018). Integrating cIEF with electrospray ionization (ESI)-MS is ideal for high-resolution separation and confident identification of proteoforms. cIEF-MS for protein characterization has been pioneered by Lee and Smith group in 1990s (Tang et al., 1995; Yang et al., 1998; Jensen et al., 1999; Paša-Tolić et al., 1999).

However, for a long time, cIEF-ESI-MS suffered from low sensitivity due to the significant sample dilution by the sheath liquid in the coaxial sheath flow CE-MS interface (Smith et al., 1988) and the ionization suppression of analytes from ampholytes. The early cIEF-MS studies required manually pulling the separation capillary out of catholyte for focusing and inserting it into the ESI interface for mobilization and MS detection (Jensen et al., 1999; Paša-Tolić et al., 1999), impeding the widespread adoption of the technique for protein characterization.

During the last two decades, great efforts have been made for improving the sensitivity of cIEF-MS through developing new CE-MS interfaces and mitigating ampholyte impacts as well as for developing automated cIEF-MS methods. Several review papers focusing on cIEF-MS have been published recently (Silvertand et al., 2008; Hühner et al., 2015; Lechner et al., 2019). In this mini review, we summarize the important technical progress of cIEF-MS and highlight most recent applications of automated cIEF-MS for top-down MS characterization of hydrophobic proteins, urinary albumin variants, charge variants of monoclonal antibodies (mAbs), and large-scale TDP of complex proteomes.

## Technical Development of cIEF-MS

### CE-MS Interface

**Figure 1:**
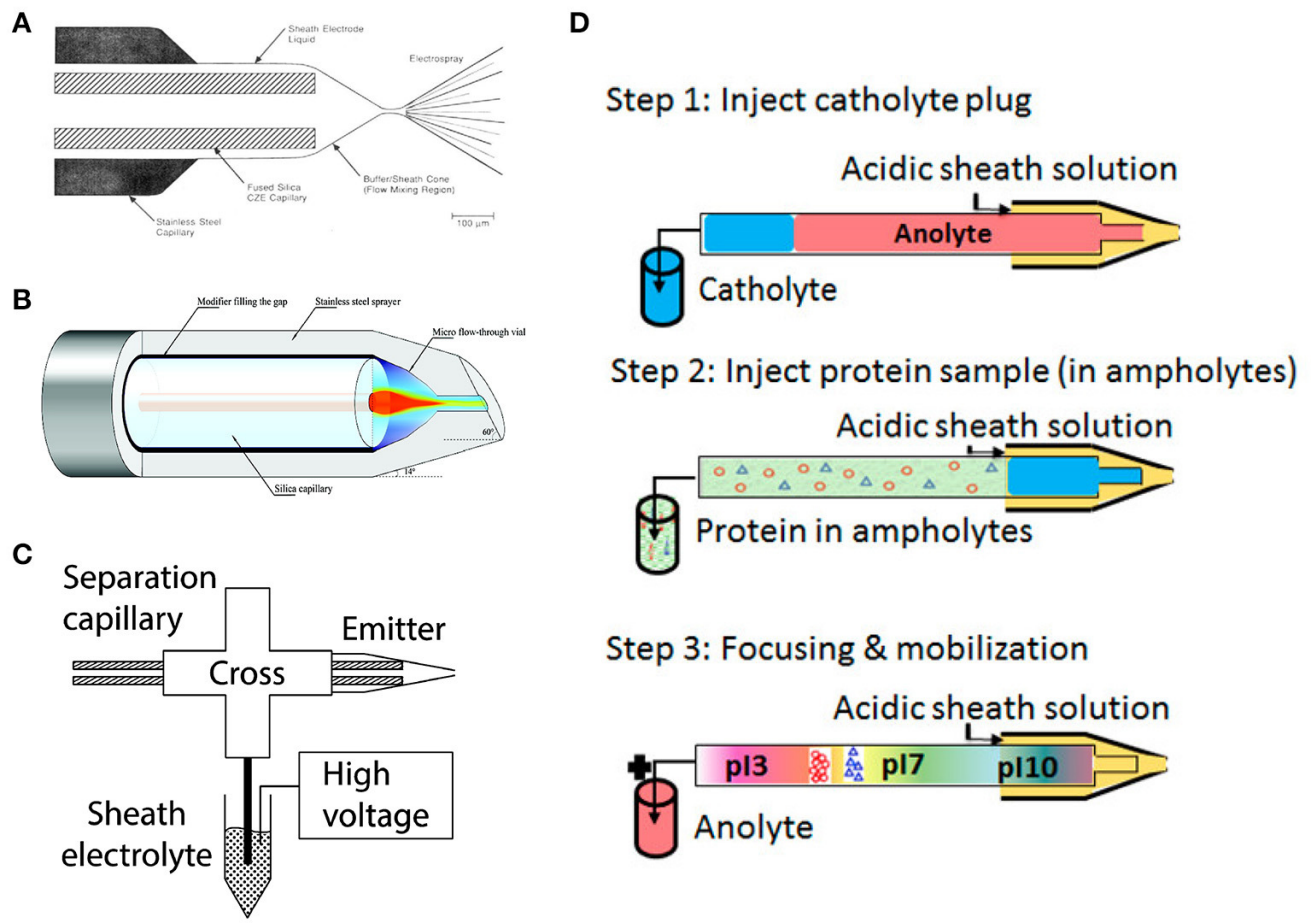
**(A)** Coaxial sheath flow interface. Reproduced with permission from Smith et al. (1988). Copyright 1988 American Chemical Society. **(B)** Flow-through microvial interface. Reproduced with permission from Zhong et al. (2011a). Copyright 2011 American Chemical Society. **(C)** Electrokinetically pumped sheath flow interface. Reproduced with permission from Sun et al. (2015). Copyright 2015 American Chemical Society. **(D)** Flowchart of automated cIEF-MS. Reproduced with permission from Dai et al. (2018). Copyright 2018 American Chemical Society.

Online hyphenation of cIEF and ESI-MS requires a CE-MS interface that can establish electrical continuity for CE separation, and meanwhile produces stable electrospray. Sheath-flow interfaces are well-suited for cIEF-MS studies. Apart from assisting ionization, the sheath liquid can serve as the chemical mobilizer to facilitate protein mobilization after cIEF focusing. The concentration of carrier ampholytes can be significantly decreased after mixing cIEF effluent with the sheath liquid, thus benefiting ESI-MS detection. The coaxial sheath flow interface (Smith et al., 1988), designed by Smith group in 1988, is the earliest version of interface used for cIEF-MS (Figure 1A). However, significant sample dilution can occur using this interface due to the much higher flow rate of the sheath liquid compared to the sample flow in CE capillary (1–10 μL/min vs. low nL/min).

Tremendous efforts have been invested to improve the sensitivity of sheath-flow interface by reducing the flow rate of sheath liquid to the nL/min level. The Chen group constructed a flow-through microvial interface by placing the separation capillary in a stainless-steel emitter (Maxwell et al., 2010) (Figure 1B). The sheath buffer is delivered through the gap between the capillary and the emitter via a springe pump at a flow rate of 100–300 nL/min. They achieved at least five-times better limit of detections (LODs) for amino acids using the interface compared to the coaxial sheath flow interface. The Dovichi group introduced an electrokinetically pumped sheath flow interface to the field in 2010 (Wojcik et al., 2010). The interface employed electroosmotic flow in the glass spray emitter to pump the sheath liquid at the nL/min level for ESI (Figure 1C). The interface was further improved regarding sensitivity and robustness by adjusting the emitter orifice size and distance between the capillary end and emitter orifice in 2013 and 2015 (Sun et al., 2013, 2015). The improved interface-based CE-MS showed extremely high sensitivity for peptides and proteoforms (Sun et al., 2013; Yang et al., 2018; Lubeckyj et al., 2019). The electrokinetically pumped sheath flow CE-MS interface has been commercialized by the CMP Scientific (https://www.cmpscientific.com/) as the EMASS-II CE-MS Ion Source. Both the two sheath-flow interfaces have been successfully applied to cIEF-MS characterization of intact proteins (Zhong et al., 2011a,b; Zhu et al., 2017). More recently, several other sheath-flow CE-MS interfaces with nL/min flow rates of sheath liquid have been developed (Choi et al., 2016; Fang et al., 2018; Krenkova et al., 2019; Höcker et al., 2020). However, based on our best knowledge, there are still no literature reports about using these new CE-MS interfaces for cIEF-MS.

### Reducing the Impact of Carrier Ampholytes

Carrier ampholytes induce ionization suppression of analytes (Dai et al., 2018), restricting the overall sensitivity of cIEF-MS. However, they are indispensable for establishing and maintaining pH gradient needed for cIEF separation. Reducing the concentration of ampholyte can significantly improve MS signal, but it adversely impacts separation resolution. Compromise has to be made between MS signal and separation resolution when performing cIEF-MS analysis and the concentration of ampholytes needs to be reduced to 0.5% or even lower (Paša-Tolić et al., 1999; Hühner et al., 2015; Zhu et al., 2017).

Besides decreasing the ampholyte concentration, several other approaches have been validated for reducing the impact of carrier ampholytes on ESI of analytes. First, integrating the cIEF with microdialysis (MD) is effective to remove the low-molecular-weight ampholytes from relatively large proteins before ESI (Lamoree et al., 1997). The method is not widely used due to reduced separation efficiency caused by the MD devices. Second, cIEF-MS has been performed in a carrier ampholyte-free condition (Zhu et al., 2012). As amino acids have amphoteric properties and are much smaller than peptides and proteins, they can be used for establishing pH gradient for cIEF separation. The drawback is amino acids cannot establish a continuous pH gradient in the separation capillary as commercialized carrier ampholytes. Third, some work has been successfully done for creating an immobilized pH gradient in cIEF capillaries by covalently immobilizing carrier ampholytes onto *in-situ* formed monolithic materials for protein separation (Zhu et al., 2006; Yang et al., 2010; Liang et al., 2011; Liu et al., 2019). However, more systematic investigations of the immobilized pH gradient cIEF-MS needs to be done before deploying it in routine protein characterization.

### Development of Automated cIEF-MS

The cIEF-MS analyses typically were implemented in a semi-online manner, where capillary outlet was inserted in a catholyte reservoir with basic buffer for focusing, and then manually transferred to an interface filled with acidic sheath liquid for mobilization and ionization (Jensen et al., 1999). Alternatively, the capillary was fixed in the interface during the whole process and sheath liquid in the interface was substituted from basic buffer to acidic sheath liquid when focusing was completed (Wang et al., 2018). The appearance of “sandwich” injection configuration in 2009 makes it possible to perform fully automated cIEF-MS analysis (Mokaddem et al., 2009). The method was carried out by filling the capillary with MS compatible catholyte buffer such as ammonia hydroxide, followed by a plug of sample-ampholyte mixture. Thus, the cIEF focusing could be facilitated after applying voltage even though its outlet was installed in an interface with acidic sheath liquid. After focusing, a low pressure (i.e., 50 mbar) or chemical mobilization was employed to drive focused proteins toward MS for detection. The chemical mobilization can automatically be initiated when cations from anolyte and anions from sheath liquid enter the capillary and gradually disrupt the pH gradient. The automated cIEF-MS method is an appealing technique for various applications.

## Applications

Because of the drastic improvement in the CE-MS interface regarding stability and sensitivity, the method for reducing the negative influence of ampholytes on ESI-MS, and the automated operation, cIEF-MS has been recognized as a powerful and sensitive analytical tool for top-down MS characterization of intact proteins.

### Automated cIEF-ESI-MS for Top-Down Characterization of Hydrophobic Proteins, Urinary Albumin, and mAb Charge Variants

Characterization of hydrophobic proteins with CE-MS is always challenging because additives for stabilizing hydrophobic proteins such as thiourea, urea, and surfactants are not compatible with MS. Mokaddem *et al*. performed automated cIEF-ESI-MS analysis of a mixture of hydrophobic and hydrophilic proteins in a glycerol-water medium with a commercialized coaxial interface (Mokaddem et al., 2009). The automated cIEF-MS was carried out in three steps (Figure 1D). First, the capillary (100 cm) was injected with a plug of catholyte (60 cm) and a plug of sample-ampholyte mixture (40 cm). Then, a voltage (30 kV) was applied on the capillary to facilitate protein focusing. Finally, after focusing completed, a pressure (50 mbar) was applied on the capillary to mobilize protein bands. The study found that glycerol in the concentration range of 10–30% (v/v) was both MS compatible and well-preserved protein solubility. The data indicate the potential of cIEF-MS for the characterization of membrane proteins. The method was later employed by Lecoeur et al. for qualitative and quantitative analysis of hydrophobic and hydrophilic whey proteins in bovine milk (Lecoeur et al., 2010).

The automated cIEF-MS with glycerol medium is also well-suited for top-down characterization of mAb charge variants. Dai *et al*. developed an automated cIEF-MS method using the similar “sandwich” injection method reported by Mokaddem et al., a capillary with neutral coating, and an electrokinetically pumped sheath flow interface (Dai et al., 2018). Using this method, the charge variants of various mAbs, including trastuzumab, bevacizumab, infliximab, and cetuximab, were well-resolved. The separation results showed good correlation with that from cIEF-UV analysis. Later, Dai et al. created a middle-up approach based on the automated cIEF-MS to boost its performance for delineating complex mAb charge variants (i.e., cetuximab), leading to the identification of at least eight different charge variants of cetuximab (Dai and Zhang, 2018). More recently, Tie et al. applied the automated cIEF-MS to the characterization of urinary albumin species from the membranous nephropathy (MN) patients (Tie et al., 2020). They observed distinct patterns of urinary albumin charge variants from the primary and secondary MN samples, suggesting the potential of the technique for distinguishing different subtypes of MN.

Wang *et al*. demonstrated top-down characterization of mAb charge variants on an automated cIEF-ESI-QTOF MS system with a flow through microvial interface (Wang et al., 2018; Wang and Chen, 2019). With consumption of only 30 ng of infliximab, four charge variants with 0.05–0.2 pI differences and 13 glycoforms were detected (Wang et al., 2018). Developing microchip-based cIEF-MS is also very attractive to pharmaceutical industry. Recent microchip-based cIEF-MS system developed by Mack et al. provided real-time optical monitoring of focusing and mobilization process of cIEF, good resolving power and high throughput (15 min each assay) for characterization of mAb charge variants (Mack et al., 2019). Besides a direct coupling of cIEF and ESI-MS, cIEF has also been coupled to CZE-MS using a mechanical valve or a nanoliter valve for high-resolution characterization of intact proteins and mAb charge variants (Hühner et al., 2017; Montealegre and Neusüß, 2018).

These works suggest automated cIEF-MS is a promising tool for quality control of therapeutic mAbs regarding charge variants and PTMs in pharmaceuticals by providing high-resolution separation and accurate mass determination. All the cIEF-MS studies mentioned here only performed protein mass measurement without MS/MS. More efforts need to be made about integrating extensive gas-phase fragmentation techniques with automated cIEF-MS for delineation of proteoforms.

### Automated cIEF-ESI-MS/MS for Large-Scale TDP

Coupling automated cIEF-MS with MS/MS enables online fragmentation of separated proteoforms for identifying proteoform sequences and localizing PTMs. Our lab presented the first work of automated cIEF-MS/MS for large-scale TDP of complex proteomes in 2020 (Xu et al., 2020). The automated cIEF-MS/MS platform was constructed by integrating a linear-polyacrylamide (LPA) coated capillary with an Orbitrap mass spectrometer via an electrokinetically pumped sheath flow interface. Based on “sandwich” injection configuration and chemical mobilization, automated cIEF-MS/MS methods identified 711 *E. coli* proteoforms in a single run by consuming only nanograms of proteins.

**Figure 2:**
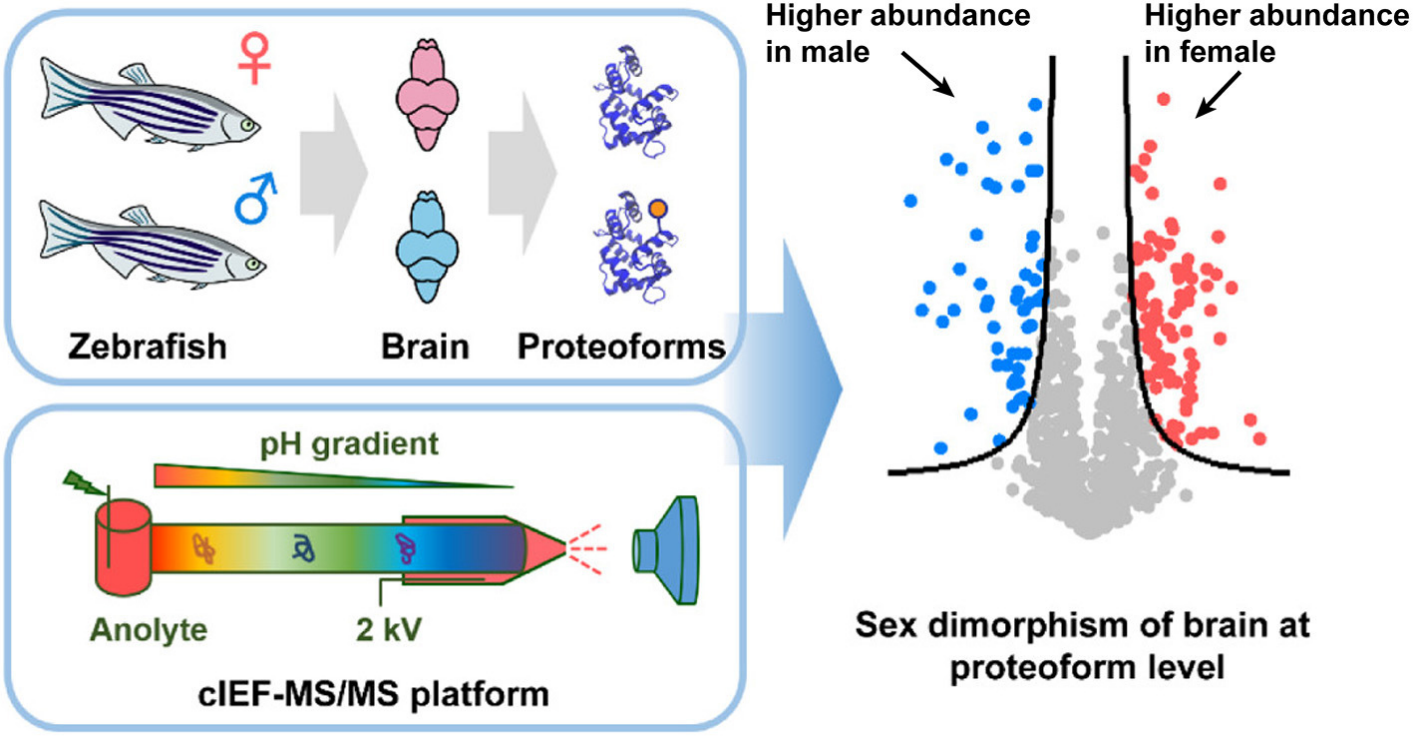
Flow chart of label-free quantitative TDP of female and male zebrafish brains using automated cIEF-MS/MS. Reproduced with permission from Xu et al. (2020). Copyright 2020 American Chemical Society.

Furthermore, combining size exclusion chromatography (SEC) and the automated cIEF-MS/MS identified nearly 2000 proteoforms from the *E. coli* proteome. SEC-cIEF-MS/MS was further employed for label-free quantitative TDP of male and female zebrafish brains (Figure 2). Thousands of proteoforms were quantified, and 263 proteoforms showed statistically significant difference in abundance between male and female zebrafish brains. Gene ontology analysis found many of these differentially expressed proteoforms were associated with neuronal development and their expression can be regulated by hormones, disclosing the sex dimorphism of zebrafish brains at the proteoform level. The work clearly demonstrates the capability of automated cIEF-MS/MS for large-scale qualitative and quantitative TDP.

## Discussion

Reversed-phase liquid chromatography (RPLC)-MS/MS is the routine choice for top-down characterization of proteoforms (Tran et al., 2011; Chen et al., 2017; Schaffer et al., 2019). However, RPLC typically fails to separate proteoforms, especially large proteoforms (i.e., larger than 30 kDa), with high separation efficiency due to their low diffusion coefficients and strong interactions with the reversed-phase beads. Capillary zone electrophoresis (CZE)-MS/MS has been suggested as a useful alternative for TDP due to high separation efficiency of CZE for proteoforms according to their electrophoretic mobilities and high sensitivity of CZE-MS for proteoform measurements (Gomes and Yates, 2019; Shen et al., 2019). Although sub-microliter sample loading volumes have been reported for TDP using CZE-MS/MS (Lubeckyj et al., 2017, 2019), the loading volume is still limited to a small portion of the total capillary volume for maintaining high separation efficiency. cIEF-MS/MS has shown high potential for advancing TDP because cIEF can achieve proteoform separations with high resolution and has much higher sample loading capacity compared to CZE.

cIEF-MS has become an important technique for top-down MS characterization of proteins. However, it still needs to overcome several technical challenges. First, current cIEF-MS methods can hardly make full use of high resolving power of cIEF. Decreasing concentration of carrier ampholytes is a common choice to enhance sensitivity, but it can cause reduced separation resolution. Immobilized pH gradient cIEF could be an alternative because it allows cIEF separation without carrier ampholytes and has shown good separation efficiency of proteins with UV detection. However, its applications in cIEF-MS still need to be systematically evaluated. Second, cIEF-MS suffers from limited lifetime of capillary coating. cIEF-MS studies generally use ammonia hydroxide (pH > 11) as catholyte. However, the most commonly used LPA coating cannot stand this high pH for a long time. Using a catholyte with pH lower than 10 can improve the stability of capillary coating (Ramsay et al., 2011). Exploring novel capillary coatings that are stable at high pH is vital for reproducible and robust cIEF-MS analyses. Third, analysis of highly basic (pI > 10) or acidic (pI < 3) proteoforms remains difficult for cIEF as the commonly used carrier ampholytes cover a pI range of 3–10. Thus, novel cIEF-MS methods are required for characterizing proteoforms with extremely low or high pIs.

## Author Contributions

TX and LS wrote the manuscript and made comments. Both authors contributed to the article and approved the submitted version.

## Conflict of Interest

The authors declare that the research was conducted in the absence of any commercial or financial relationships that could be construed as a potential conflict of interest.
